# 3-(3-Methyl­phen­yl)-5-(quinolin-8-yl­meth­oxy)-1,2,4-oxa­diazole monohydrate

**DOI:** 10.1107/S160053681302477X

**Published:** 2013-09-12

**Authors:** Lu Yang, Wei Liu, Han Wang, Xing-Wei Chen, Hai-Bo Wang

**Affiliations:** a College of Science, Nanjing University of Technology, Xinmofan Road No.5 Nanjing, Nanjing 210009, People’s Republic of China; b College of Food Science and Light Industry, Nanjing University of Technology, Xinmofan Road No.5 Nanjing, Nanjing 210009, People’s Republic of China

## Abstract

In the title compound, C_19_H_15_N_3_O_2_·H_2_O, the oxa­diazole ring and the quinoline unit are almost coplanar, making a dihedral angle of 7.66 (8)°. The dihedral angle between the benzene ring and the quinoline system is 25.95 (8)° while that between the benzene and the oxa­diazole rings is 18.88 (9)°. The water mol­ecule is hydrogen bonded to an oxa­diazole N atom and to the quinoline N atom. In the crystal, these units are linked *via* C—H⋯O hydrogen bonds, forming two-dimensional net­works lying parallel to the *ab* plane.

## Related literature

For the preparation of the title compound, see: Chiou & Shine (1989 ▶). For the biological activity of 1,2,4-oxa­diazole derivatives, see: Street *et al.* (1990 ▶). For metal complexes of related compounds, see: da Silva *et al.* (1999 ▶); Pibiri *et al.* (2010 ▶); Terenzi *et al.* (2011 ▶). For bond-length data, see: Allen *et al.* (1987 ▶). 
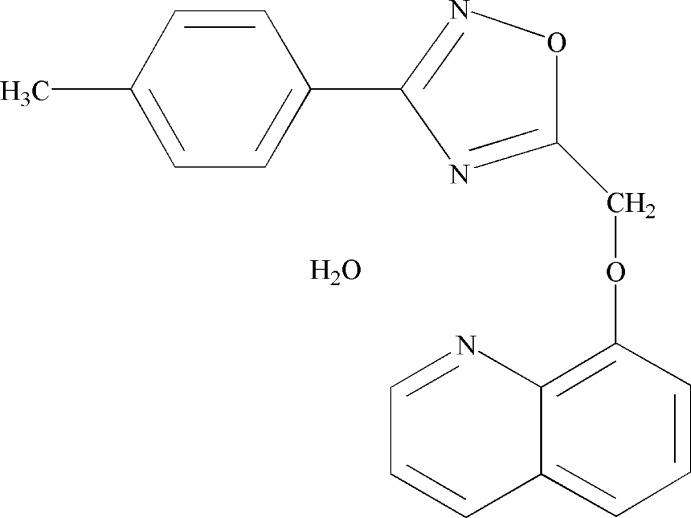


## Experimental

### 

#### Crystal data

C_19_H_15_N_3_O_2_·H_2_O
*M* *_r_* = 335.36
Triclinic, 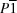

*a* = 7.2070 (14) Å
*b* = 7.6200 (15) Å
*c* = 15.109 (3) Å
α = 92.62 (3)°
β = 90.19 (3)°
γ = 92.15 (3)°
*V* = 828.3 (3) Å^3^
*Z* = 2
Mo *K*α radiation
μ = 0.09 mm^−1^
*T* = 293 K
0.30 × 0.10 × 0.10 mm

#### Data collection

Enraf–Nonius CAD-4 diffractometer
Absorption correction: ψ scan (North *et al.*, 1968 ▶) *T*_min_ = 0.973, *T*_max_ = 0.991
3302 measured reflections
3039 independent reflections
1949 reflections with *I* > 2σ(*I*)
*R*_int_ = 0.015
3 standard reflections every 200 reflections intensity decay: 1%

#### Refinement

*R*[*F* ^2^ > 2σ(*F* ^2^)] = 0.054
*wR*(*F* ^2^) = 0.162
*S* = 1.00
3039 reflections
233 parameters
H atoms treated by a mixture of independent and constrained refinement
Δρ_max_ = 0.24 e Å^−3^
Δρ_min_ = −0.18 e Å^−3^

### 

Data collection: *CAD-4 EXPRESS* (Enraf–Nonius, 1994 ▶); cell refinement: *CAD-4 EXPRESS*; data reduction: *XCAD4* (Harms & Wocadlo, 1995 ▶); program(s) used to solve structure: *SHELXS97* (Sheldrick, 2008 ▶); program(s) used to refine structure: *SHELXL97* (Sheldrick, 2008 ▶); molecular graphics: *ORTEP-3 for Windows* (Farrugia, 2012 ▶); software used to prepare material for publication: *publCIF* (Westrip, 2010 ▶).

## Supplementary Material

Crystal structure: contains datablock(s) D, I. DOI: 10.1107/S160053681302477X/im2428sup1.cif

Structure factors: contains datablock(s) I. DOI: 10.1107/S160053681302477X/im2428Isup2.hkl

Additional supplementary materials:  crystallographic information; 3D view; checkCIF report

## Figures and Tables

**Table 1:** Hydrogen-bond geometry (Å, °)

*D*—H⋯*A*	*D*—H	H⋯*A*	*D*⋯*A*	*D*—H⋯*A*
O*W*—H*WB*⋯N2	0.91 (3)	2.09 (3)	2.980 (3)	169 (3)
O*W*—H*WA*⋯N1	0.94 (3)	1.91 (3)	2.830 (3)	165 (3)
C7—H7*A*⋯O*W* ^i^	0.93	2.51	3.272 (3)	139
C10—H10*A*⋯O*W* ^ii^	0.97	2.55	3.482 (3)	160
C10—H10*B*⋯O*W* ^iii^	0.97	2.59	3.534 (3)	164

Symmetry codes: (i) 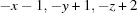; (ii) 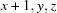; (iii) 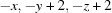.

**Fig. 1.**
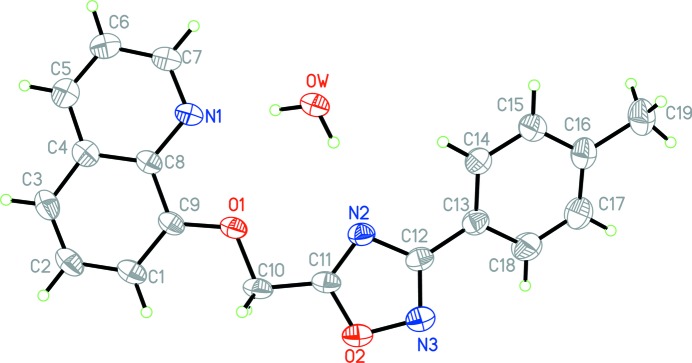
Molecular structure of the title molecule with displacement ellipsoids drawn at the 50% probability level.

**Fig. 2.**
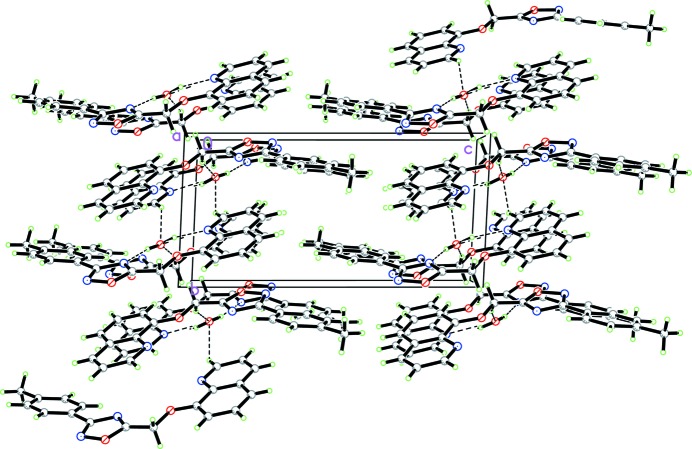
Packing diagram of the title compound. Hydrogen bonds are shown as dashed lines.

## supplementary crystallographic information

### 1. Comment

1,2,4-Oxadiazole derivatives have shown high biological activity, such as antibacterial, anti-HIV and weed control (Street *et al.*, 1990). They are therefore widely used in medicinal chemistry and as pesticides. 1,2,4-Oxadiazole derivatives in combination with metal ions can also be used in fluorescent recognition (da Silva *et al.*, 1999; Pibiri *et al.*, 2010; Terenzi *et al.*, 2011). The title compound 5-(quinoline-8-ylmethoxy)-3-p-tolyl-1,2,4-oxadiazole was also used in metal ions fluorescent recognition. In the molecule of 5-(quinoline-8-ylmethoxy)-3-p-tolyl-1,2,4-oxadiazole hydrate (Fig. 1) bond lengths (Allen *et al.*, 1987) and angles are within normal ranges.The oxadiazol ring and the quinoline moiety are almost coplanar showing a dihedral angle of 7.66 (8)°. The dihedral angles between the benzene ring and the quinoline system is 25.95 (8)°, the corresponding angle between the benzene and the oxadiazol rings is 18.88 (9)°. The crystal structure is established by intermolecular N—H···O and O—H···O hydrogen bonds (Fig. 2).

### 2. Experimental

5-(Quinoline-8-ylmethoxy)-3-p-tolyl-1,2,4-oxadiazole was prepared by a literature method (Chiou & Shine, 1989). 3-(4-Methyl-phenyl)-5-chloromethyl-1,2,4-oxadizole (3.4 g, 16.4 mmol), 8-hydroxy-quinoline (2.4 g, 16.4 mmol), potassium carbonate (3.4 g ,24.6 mmol) and potassium iodide (catalytic amount) were added to acetone (40 ml). The mixture was then heated to reflux for 6 hours. After being cooled to room temperature, the mixture was filtered and evaporated to afford the product as a yellow solid. The crude product was re-crystallized from ethyl acetate (yield 3.1g, 59.8%). Crystals suitable for X-ray analysis were obtained by slow evaporation of an ethyl acetate solution.

### 3. Refinement

H atoms were positioned geometrically, with C-H = 0.93, 0.97 and 0.96 Å for aromatic, methylene and methyl H, respectively, and constrained to ride on their parent atoms, with U_iso_(H) = xU_eq_(C,N), where x = 1.5 for methyl H and x = 1.2 for all other H atoms. Hydrogen atoms of the solvent water molecule have been determined from Fourier maps and refined freely.

### Crystal data

**Table:**

C_19_H_15_N_3_O_2_·H_2_O	*Z* = 2
*M**_r_* = 335.36	*F*(000) = 352
Triclinic, *P*1	*D*_x_ = 1.345 Mg m^−^^3^
Hall symbol: -P 1	Melting point: 342 K
*a* = 7.2070 (14) Å	Mo *K*α radiation, λ = 0.71073 Å
*b* = 7.6200 (15) Å	Cell parameters from 25 reflections
*c* = 15.109 (3) Å	θ = 9–13°
α = 92.62 (3)°	µ = 0.09 mm^−^^1^
β = 90.19 (3)°	*T* = 293 K
γ = 92.15 (3)°	Block, yellow
*V* = 828.3 (3) Å^3^	0.30 × 0.10 × 0.10 mm

### Data collection

**Table:**

Enraf–Nonius CAD-4 diffractometer	1949 reflections with *I* > 2σ(*I*)
Radiation source: fine-focus sealed tube	*R*_int_ = 0.015
Graphite monochromator	θ_max_ = 25.4°, θ_min_ = 1.4°
ω/2θ scans	*h* = 0→8
Absorption correction: ψ scan (North *et al.*, 1968)	*k* = −9→9
*T*_min_ = 0.973, *T*_max_ = 0.991	*l* = −18→18
3302 measured reflections	3 standard reflections every 200 reflections
3039 independent reflections	intensity decay: 1%

### Refinement

**Table:**

Refinement on *F*^2^	Secondary atom site location: difference Fourier map
Least-squares matrix: full	Hydrogen site location: inferred from neighbouring sites
*R*[*F*^2^ > 2σ(*F*^2^)] = 0.054	H atoms treated by a mixture of independent and constrained refinement
*wR*(*F*^2^) = 0.162	*w* = 1/[σ^2^(*F*_o_^2^) + (0.097*P*)^2^] where *P* = (*F*_o_^2^ + 2*F*_c_^2^)/3
*S* = 1.00	(Δ/σ)_max_ < 0.001
3039 reflections	Δρ_max_ = 0.24 e Å^−^^3^
233 parameters	Δρ_min_ = −0.18 e Å^−^^3^
0 restraints	Extinction correction: *SHELXL97* (Sheldrick, 2008), Fc^*^=kFc[1+0.001xFc^2^λ^3^/sin(2θ)]^-1/4^
Primary atom site location: structure-invariant direct methods	Extinction coefficient: 0.033 (7)

### Special details

**Table:**

Geometry. All esds (except the esd in the dihedral angle between two l.s. planes) are estimated using the full covariance matrix. The cell esds are taken into account individually in the estimation of esds in distances, angles and torsion angles; correlations between esds in cell parameters are only used when they are defined by crystal symmetry. An approximate (isotropic) treatment of cell esds is used for estimating esds involving l.s. planes.
Refinement. Refinement of F^2^ against ALL reflections. The weighted R-factor wR and goodness of fit S are based on F^2^, conventional R-factors R are based on F, with F set to zero for negative F^2^. The threshold expression of F^2^ > 2sigma(F^2^) is used only for calculating R-factors(gt) etc. and is not relevant to the choice of reflections for refinement. R-factors based on F^2^ are statistically about twice as large as those based on F, and R- factors based on ALL data will be even larger.

### Fractional atomic coordinates and isotropic or equivalent isotropic displacement parameters (Å^2^)

**Table:**

	*x*	*y*	*z*	*U*_iso_*/*U*_eq_	
O1	0.0819 (2)	0.7951 (2)	0.99656 (11)	0.0571 (5)	
N1	−0.1889 (3)	0.6301 (2)	1.08273 (13)	0.0522 (5)	
C1	0.2813 (3)	0.8077 (3)	1.12657 (18)	0.0596 (7)	
H1B	0.3763	0.8670	1.0974	0.071*	
N2	0.0057 (3)	0.8438 (2)	0.81930 (13)	0.0511 (5)	
O2	0.2873 (2)	0.9553 (2)	0.80096 (12)	0.0695 (6)	
C2	0.3046 (4)	0.7675 (4)	1.21478 (19)	0.0667 (7)	
H2B	0.4155	0.8005	1.2437	0.080*	
C3	0.1700 (4)	0.6819 (4)	1.25913 (18)	0.0656 (7)	
H3A	0.1894	0.6553	1.3178	0.079*	
N3	0.2016 (3)	0.9460 (3)	0.71676 (15)	0.0714 (7)	
C4	−0.0008 (3)	0.6320 (3)	1.21701 (17)	0.0551 (6)	
C5	−0.1489 (4)	0.5465 (3)	1.25918 (18)	0.0642 (7)	
H5A	−0.1374	0.5179	1.3181	0.077*	
C6	−0.3092 (4)	0.5051 (3)	1.21418 (19)	0.0653 (7)	
H6A	−0.4084	0.4483	1.2416	0.078*	
C7	−0.3218 (3)	0.5497 (3)	1.12629 (18)	0.0594 (7)	
H7A	−0.4323	0.5206	1.0962	0.071*	
C8	−0.0273 (3)	0.6721 (3)	1.12723 (16)	0.0475 (6)	
C9	0.1196 (3)	0.7605 (3)	1.08256 (16)	0.0486 (6)	
C10	0.2240 (3)	0.8842 (3)	0.94993 (17)	0.0557 (6)	
H10A	0.3386	0.8219	0.9528	0.067*	
H10B	0.2460	1.0022	0.9757	0.067*	
C11	0.1608 (3)	0.8911 (3)	0.85698 (17)	0.0511 (6)	
C12	0.0369 (3)	0.8783 (3)	0.73185 (16)	0.0535 (6)	
C13	−0.0981 (3)	0.8373 (3)	0.66076 (16)	0.0553 (6)	
C14	−0.2843 (4)	0.8113 (3)	0.67896 (17)	0.0617 (7)	
H14A	−0.3249	0.8161	0.7374	0.074*	
C15	−0.4105 (4)	0.7784 (4)	0.61087 (18)	0.0687 (7)	
H15A	−0.5356	0.7628	0.6245	0.082*	
C16	−0.3573 (4)	0.7677 (3)	0.52325 (17)	0.0677 (8)	
C17	−0.1699 (5)	0.7904 (4)	0.50615 (19)	0.0847 (9)	
H17A	−0.1291	0.7826	0.4478	0.102*	
C18	−0.0424 (4)	0.8242 (4)	0.57307 (19)	0.0814 (9)	
H18A	0.0828	0.8385	0.5594	0.098*	
C19	−0.4974 (5)	0.7358 (4)	0.45006 (19)	0.0905 (10)	
H19A	−0.4352	0.7339	0.3940	0.136*	
H19B	−0.5845	0.8282	0.4523	0.136*	
H19C	−0.5622	0.6250	0.4571	0.136*	
OW	−0.3313 (2)	0.7299 (3)	0.91840 (14)	0.0733 (6)	
HWB	−0.236 (5)	0.757 (4)	0.8819 (19)	0.088*	
HWA	−0.274 (4)	0.682 (4)	0.967 (2)	0.088*	

### Atomic displacement parameters (Å^2^)

**Table:**

	*U*^11^	*U*^22^	*U*^33^	*U*^12^	*U*^13^	*U*^23^
O1	0.0383 (8)	0.0683 (11)	0.0639 (11)	−0.0106 (8)	−0.0065 (8)	0.0075 (8)
N1	0.0400 (10)	0.0500 (11)	0.0661 (13)	−0.0038 (9)	−0.0033 (10)	0.0013 (9)
C1	0.0387 (13)	0.0624 (16)	0.0769 (19)	0.0010 (11)	−0.0102 (12)	−0.0030 (13)
N2	0.0425 (11)	0.0550 (12)	0.0551 (12)	−0.0019 (9)	0.0074 (9)	−0.0021 (9)
O2	0.0450 (10)	0.0884 (13)	0.0744 (13)	−0.0133 (9)	0.0078 (9)	0.0081 (10)
C2	0.0498 (15)	0.0751 (18)	0.0742 (19)	0.0041 (13)	−0.0197 (14)	−0.0088 (14)
C3	0.0634 (17)	0.0752 (18)	0.0579 (16)	0.0075 (14)	−0.0165 (14)	−0.0034 (13)
N3	0.0556 (13)	0.0913 (17)	0.0670 (15)	−0.0103 (12)	0.0084 (12)	0.0106 (12)
C4	0.0528 (14)	0.0486 (13)	0.0640 (16)	0.0094 (11)	−0.0032 (12)	−0.0022 (11)
C5	0.0694 (18)	0.0598 (16)	0.0643 (17)	0.0119 (14)	0.0029 (14)	0.0052 (13)
C6	0.0580 (16)	0.0608 (16)	0.0773 (19)	−0.0011 (13)	0.0091 (14)	0.0079 (14)
C7	0.0457 (14)	0.0567 (15)	0.0753 (18)	−0.0028 (12)	−0.0016 (13)	0.0017 (13)
C8	0.0405 (12)	0.0420 (12)	0.0595 (15)	0.0032 (10)	−0.0053 (11)	−0.0046 (10)
C9	0.0370 (12)	0.0478 (13)	0.0607 (15)	0.0047 (10)	−0.0081 (11)	−0.0021 (11)
C10	0.0327 (11)	0.0617 (15)	0.0713 (17)	−0.0062 (11)	0.0050 (11)	−0.0053 (12)
C11	0.0351 (12)	0.0505 (13)	0.0668 (16)	−0.0005 (10)	0.0058 (11)	−0.0041 (11)
C12	0.0478 (13)	0.0534 (14)	0.0592 (16)	0.0016 (11)	0.0118 (11)	0.0019 (11)
C13	0.0573 (15)	0.0544 (14)	0.0540 (15)	0.0009 (12)	0.0074 (12)	0.0002 (11)
C14	0.0577 (15)	0.0757 (17)	0.0516 (15)	0.0001 (13)	0.0060 (12)	0.0021 (12)
C15	0.0590 (15)	0.0846 (19)	0.0623 (18)	−0.0007 (14)	−0.0003 (13)	0.0034 (14)
C16	0.085 (2)	0.0642 (17)	0.0536 (17)	0.0042 (15)	−0.0039 (14)	0.0020 (13)
C17	0.090 (2)	0.112 (3)	0.0512 (17)	−0.0007 (19)	0.0109 (16)	−0.0037 (16)
C18	0.0700 (18)	0.111 (2)	0.0616 (18)	−0.0049 (17)	0.0182 (16)	−0.0037 (16)
C19	0.111 (3)	0.100 (2)	0.0606 (18)	0.003 (2)	−0.0170 (18)	0.0006 (17)
OW	0.0446 (10)	0.1012 (15)	0.0735 (13)	−0.0152 (10)	−0.0081 (9)	0.0147 (11)

### Geometric parameters (Å, º)

**Table:**

O1—C9	1.366 (3)	C7—H7A	0.9300
O1—C10	1.415 (3)	C8—C9	1.422 (3)
N1—C7	1.313 (3)	C10—C11	1.479 (4)
N1—C8	1.363 (3)	C10—H10A	0.9700
C1—C9	1.368 (3)	C10—H10B	0.9700
C1—C2	1.392 (4)	C12—C13	1.462 (3)
C1—H1B	0.9300	C13—C14	1.380 (4)
N2—C11	1.285 (3)	C13—C18	1.386 (4)
N2—C12	1.376 (3)	C14—C15	1.379 (4)
O2—C11	1.340 (3)	C14—H14A	0.9300
O2—N3	1.410 (3)	C15—C16	1.379 (4)
C2—C3	1.346 (4)	C15—H15A	0.9300
C2—H2B	0.9300	C16—C17	1.381 (4)
C3—C4	1.415 (3)	C16—C19	1.501 (4)
C3—H3A	0.9300	C17—C18	1.373 (4)
N3—C12	1.301 (3)	C17—H17A	0.9300
C4—C5	1.400 (4)	C18—H18A	0.9300
C4—C8	1.418 (3)	C19—H19A	0.9600
C5—C6	1.358 (4)	C19—H19B	0.9600
C5—H5A	0.9300	C19—H19C	0.9600
C6—C7	1.389 (4)	OW—HWB	0.91 (3)
C6—H6A	0.9300	OW—HWA	0.94 (3)
			
C9—O1—C10	116.60 (18)	O1—C10—H10B	110.3
C7—N1—C8	117.4 (2)	C11—C10—H10B	110.3
C9—C1—C2	120.5 (2)	H10A—C10—H10B	108.5
C9—C1—H1B	119.8	N2—C11—O2	113.4 (2)
C2—C1—H1B	119.8	N2—C11—C10	131.4 (2)
C11—N2—C12	103.1 (2)	O2—C11—C10	115.16 (19)
C11—O2—N3	105.97 (17)	N3—C12—N2	114.0 (2)
C3—C2—C1	121.4 (2)	N3—C12—C13	122.2 (2)
C3—C2—H2B	119.3	N2—C12—C13	123.9 (2)
C1—C2—H2B	119.3	C14—C13—C18	118.2 (3)
C2—C3—C4	120.4 (3)	C14—C13—C12	121.0 (2)
C2—C3—H3A	119.8	C18—C13—C12	120.8 (2)
C4—C3—H3A	119.8	C15—C14—C13	120.2 (2)
C12—N3—O2	103.59 (19)	C15—C14—H14A	119.9
C5—C4—C3	123.8 (3)	C13—C14—H14A	119.9
C5—C4—C8	117.1 (2)	C14—C15—C16	122.2 (3)
C3—C4—C8	119.0 (2)	C14—C15—H15A	118.9
C6—C5—C4	120.1 (3)	C16—C15—H15A	118.9
C6—C5—H5A	120.0	C15—C16—C17	116.9 (3)
C4—C5—H5A	120.0	C15—C16—C19	121.3 (3)
C5—C6—C7	118.5 (3)	C17—C16—C19	121.8 (3)
C5—C6—H6A	120.7	C18—C17—C16	121.7 (3)
C7—C6—H6A	120.7	C18—C17—H17A	119.2
N1—C7—C6	124.6 (2)	C16—C17—H17A	119.2
N1—C7—H7A	117.7	C17—C18—C13	120.8 (3)
C6—C7—H7A	117.7	C17—C18—H18A	119.6
N1—C8—C4	122.3 (2)	C13—C18—H18A	119.6
N1—C8—C9	119.1 (2)	C16—C19—H19A	109.5
C4—C8—C9	118.7 (2)	C16—C19—H19B	109.5
O1—C9—C1	125.1 (2)	H19A—C19—H19B	109.5
O1—C9—C8	114.81 (19)	C16—C19—H19C	109.5
C1—C9—C8	120.1 (2)	H19A—C19—H19C	109.5
O1—C10—C11	107.17 (18)	H19B—C19—H19C	109.5
O1—C10—H10A	110.3	HWB—OW—HWA	104 (3)
C11—C10—H10A	110.3		
			
C9—C1—C2—C3	0.0 (4)	C12—N2—C11—O2	0.9 (3)
C1—C2—C3—C4	−0.8 (4)	C12—N2—C11—C10	−177.9 (2)
C11—O2—N3—C12	−0.1 (3)	N3—O2—C11—N2	−0.5 (3)
C2—C3—C4—C5	−178.5 (2)	N3—O2—C11—C10	178.5 (2)
C2—C3—C4—C8	0.6 (4)	O1—C10—C11—N2	5.4 (4)
C3—C4—C5—C6	179.2 (2)	O1—C10—C11—O2	−173.34 (19)
C8—C4—C5—C6	0.1 (4)	O2—N3—C12—N2	0.7 (3)
C4—C5—C6—C7	0.0 (4)	O2—N3—C12—C13	−177.1 (2)
C8—N1—C7—C6	0.1 (4)	C11—N2—C12—N3	−1.0 (3)
C5—C6—C7—N1	−0.1 (4)	C11—N2—C12—C13	176.7 (2)
C7—N1—C8—C4	0.0 (3)	N3—C12—C13—C14	−162.3 (3)
C7—N1—C8—C9	−179.5 (2)	N2—C12—C13—C14	20.1 (4)
C5—C4—C8—N1	−0.1 (3)	N3—C12—C13—C18	17.4 (4)
C3—C4—C8—N1	−179.2 (2)	N2—C12—C13—C18	−160.1 (3)
C5—C4—C8—C9	179.4 (2)	C18—C13—C14—C15	−1.8 (4)
C3—C4—C8—C9	0.3 (3)	C12—C13—C14—C15	177.9 (2)
C10—O1—C9—C1	1.1 (3)	C13—C14—C15—C16	0.9 (4)
C10—O1—C9—C8	179.53 (19)	C14—C15—C16—C17	0.4 (4)
C2—C1—C9—O1	179.3 (2)	C14—C15—C16—C19	−178.6 (3)
C2—C1—C9—C8	0.9 (4)	C15—C16—C17—C18	−0.7 (5)
N1—C8—C9—O1	0.0 (3)	C19—C16—C17—C18	178.2 (3)
C4—C8—C9—O1	−179.54 (19)	C16—C17—C18—C13	−0.2 (5)
N1—C8—C9—C1	178.5 (2)	C14—C13—C18—C17	1.5 (5)
C4—C8—C9—C1	−1.1 (3)	C12—C13—C18—C17	−178.2 (3)
C9—O1—C10—C11	174.34 (18)		

### Hydrogen-bond geometry (Å, º)

**Table:**

*D*—H···*A*	*D*—H	H···*A*	*D*···*A*	*D*—H···*A*
O*W*—H*WB*···N2	0.91 (3)	2.09 (3)	2.980 (3)	169 (3)
O*W*—H*WA*···N1	0.94 (3)	1.91 (3)	2.830 (3)	165 (3)
C7—H7*A*···O*W*^i^	0.93	2.51	3.272 (3)	139
C10—H10*A*···O*W*^ii^	0.97	2.55	3.482 (3)	160
C10—H10*B*···O*W*^iii^	0.97	2.59	3.534 (3)	164

Symmetry codes: (i) −*x*−1, −*y*+1, −*z*+2; (ii) *x*+1, *y*, *z*; (iii) −*x*, −*y*+2, −*z*+2.
